# Parasitic Amoebae of Man

**Published:** 1912-08

**Authors:** 


Parasitic Amoebae of Man.—Craig (*New Orleans Medical and Surgical Journal,* July, 1912) states that at least two amebae parasitic in man are capable of producing dysentery—the Entameba tetragena and the Entameba histolytica—and one species—the Entameba coli—commonly observed in both healthy and diseased individuals, is a harmless commensal of the human intestine.

Any physician who has had proper laboratory training should be able to differentiate these species after the careful study of a few cases.

The E. coli forms systs containing 8 daughter nuclei—sometimes more; E. tetragena cysts contain 4 daughter nuclei; while the E. histolytica forms minute spores which are budded from the parent organism.

When it is considered that from 30-50% of healthy individuals in the tropics and subtropics show the harmless E. coli in the feces, the importance of recognizing the parasite before making a diagnosis of amebic dysentery is apparent.

